# A review of the omicrine genera *Omicrogiton*, *Mircogioton* and *Peratogonus* of China (Coleoptera, Hydrophilidae, Sphaeridiinae)

**DOI:** 10.3897/zookeys.511.8980

**Published:** 2015-07-02

**Authors:** Fenglong Jia, Renchao Lin, Bijun Li, Martin Fikáček

**Affiliations:** 1 Institute of Entomology, Sun Yat-sen University, Guangzhou, 510275, Guangdong, China; 2 Department of Entomology, National Museum, Cirkusová 1740, CZ-193 00 Praha 9 – Horní Počernice, Czech Republic; 3 Department of Zoology, Faculty of Science, Charles University in Prague, Viničná 7, CZ-128 44 Praha 2, Czech Republic

**Keywords:** Hydrophilidae, Sphaeridiinae, Omicrini, *Omicrogiton*, *Mircogioton*, *Peratogonus*, new species, new synonym, new record, Oriental region, China

## Abstract

The Chinese species of the genera *Omicrogiton* Orchymont, 1919, *Peratogonus* Sharp, 1884 and *Mircogioton* Orchymont, 1937 are reviewed, diagnosed and keyed. *Mircogioton* and *Omicrogiton* are reported for the first time from China, *Peratogonus* for the first time for mainland China. Five species are recognized: *Omicrogiton coomani* Balfour-Browne, 1939 (Guangdong, Hongkong), *Omicrogiton hainanensis* **sp. n.** (Hainan), *Omicrogiton roberti* **sp. n.** (Hainan), *Mircogioton coomani* Orchymont, 1937 (Yunnan), and *Peratogonus reversus* Sharp, 1884 (Guangdong, Jiangxi, Taiwan). Lectotype of *Omicrogiton coomani* is designated. *Mircogioton cognitus* (Malcolm, 1981), **syn. n.** is considered a junior subjective synonym of *Mircogioton coomani* Orchymont, 1939. Species of *Mircogioton* and *Omicrogiton* inhabit decaying banana trunks, whereas *Peratogonus reversus* was always collected from moist forest leaf litter.

## Introduction

A total of 15 genera and 104 species of the tribe Omicrini Smetana, 1975 have been described world-wide (Hansen 1999; Short and Fikáček 2011, 2013). Eleven of these genera may be found in the Oriental Region: *Oreomicrus* Malcolm, 1980, *Tylomicrus* Schödl, 1995, *Nannomicrus* Bameul, 1991, *Litrosurus* Orchymont, 1925, *Stanmalcolmia* Bameul, 1993, and *Mircogioton* Orchymont, 1937 are endemic to the Oriental Region, *Peratogonus* Sharp, 1884 and *Noteropagus* Orchymont, 1919 are principally Oriental but also reach the Palaearctic or Pacific Regions, respectively. *Paromicrus* Scott, 1913 and *Psalitrus* Orchymont, 1919 occur in the Oriental and Afrotropical Regions (Africa), and *Aculomicrus* Smetana, 1990 that occurs in the Neotropic Region reaches the Oriental Region only in the Malay Archipelago (and this Bornean species may actually belong to a different genus: Fikáček 2010). Only two species of the tribe were so far recorded from China, in both cases from Taiwan: *Peratogonus reversus* Sharp, 1884 by Knisch (1921) and *Psalitrus sauteri* Orchymont, 1929 by Orchymont (1929). Not a single species of the tribe was so far recorded from the mainland China.

Since 2009, a lot of material of the tribe Omicrini was collected by us or our colleagues in various parts of Southern China, confirming that at least six omicrine genera occur in mainland China and/or Taiwan: *Psalitrus*, *Noteropagus*, *Paromicrus*, *Peratogonus*, *Omicrogiton* and *Mircogioton*. The latter three genera are revised in this contribution, in which we are providing diagnoses, identification keys and biology data of five species, of which two are described as new.

## Material and methods

Male genitalia were dissected in a portion of specimens of each species. In specimens deposited in SYSU, dissected genitalia was transferred to a drop of absolute alcohol for removing membranes after 8-10 hours in 10% KOH at room temperature, and subsequently mounted into a drop of glycerine on a piece of transparent plastic slide attached below the respective specimens. In specimens deposited in NMPC and in the holotype of *Omicrogiton hainanensis* sp. n., the dissected male genitalia were mounted into a drop of alcohol-soluble Euparal resin on a piece of glass attached below the respective specimens. Specimens from BMNH were dissected by R. B. Angus, the genitalia were placed without any additional treatment into a water-soluble dimethyl hydantoin formaldehyde resin on the same card as the beetle. Male genitalia and morphological characters were examined using a Nikon SMZ800 compound microscope. Genitalia photographs were taken using a Zeiss Axioskop 40 or Olympus BX41 compound microscopes and combined with AutoMontage or Helicon Focus software, respectively. Photographs of habitus and external morphology were taken using a Leica M205C stereomicroscope and combined with AutoMontage software.

Detailed descriptions of the tribe Omicrini and the genera treated in this study were provided by Hansen (1991). Morphological terminology largely follows Hansen (1991) and Komarek (2004), classification follows Short and Fikáček (2013).

Examined specimens are deposited in the following collections:

AFCD Agriculture, Fisheries and Conservation Department, Hong Kong;

BMNH Natural History Museum, London;

IRSN Institute Royal de Sciences naturelles, Brussels, Belgium;

IZCAS Chinese Academy of Sciences, Institute of Zoology, Beijing, China;

NMPC National Museum, Prague, Czech Republic;

MNHG Museum d’Histoire naturelle, Genève, Switzerland;

SYSU Entomological Collection of Sun Yat-sun University, Guangzhou, China.

For comparative reasons, we have examined also the following material of *Omicrogiton* species not occurring in China:

*Omicrogiton gomyi* Bameul, 1986: Holotype: male (MNHG): La Réunion / Takamaka 26-I-78 / chemin du Barrage / tamisage souche très humide / Y Gomy // male symbol // HOLOTYPE // *Omicrogiton* / *gomyi* n. sp. / HOLOTYPE / F. BAMEUL det. 1985. The specimen is dissected and its genitalia were probably mounted in a drop of dimethyl hydantoin formaldehyde resin, which is still present on a piece of transparent plastic below the specimen. However, we failed to find any genitalia in this drop – either they were never placed there, or they became completely transparent due to the long-term effect of dimethyl hydantoin formaldehyde resin. We were therefore not able to compare the genital morphology of this species with that of *Omicrogiton hainanensis* sp. n., as originally planned. New material from Reunion Island is necessary to perform this detailed comparison.

*Omicrogiton insularis* Orchymont, 1919: Syntype: 1 female (IRSN): Engamo / Bua-Bua V.-VI. / Modigliani 1891 // Coll. A. d’Orchymont // Para- / type // A. d’Orchymont det / *Omicrogiton* / *insularis* Orch. / Cotype. Additional specimens: 1 male (NMPC): Sarawak, Kapit distr., Sebong, Baleh riv., 6-21.iii.1994, Sv. Bílý lgt.; 1 male, 2 females, 1 unsexed specimen (NMPC): Solomon Islands, Guadalcanal, Mt. Austine – Barana vill. env. (gardens, in rotten *Musa*), 9°28.0'S 159°58.4'E, 280 m, 23.xi.–8.xii.2013, Jiří Hájek lgt.

### Key to Chinese Omicrini

The following key allows to identify all genera of the tribe Omicrini occurring in China based on our published and unpublished data, and all species of the genus *Omicrogiton* based on the revision performed in this paper. The generic key is adapted from that of Hansen (1991).

**Table:** 

1	Antenna with 8 antennomeres, antennal club loosely segmented. Mesoventral plate slightly wider than long, subpentagonal, contacting metaventral process	***Psalitrus* Orchymont, 1919**
	(more species known from China, to be revised by the authors)	
–	Antenna with 9 antennomeres, antennal club compact. Mesoventral plate either wider than long, longer than wide, or distinctly isolated from metaventrite	**2**
2	First ventrite not carinate medially. Mesoventral plate narrowly elongate	**3**
–	First ventrite carinate medially. Mesoventral plate broadly pentagonal	**4**
3	Mesoventral plate fused with metaventral process, forming a common meso-metaventral keel. First metatarsomere much longer than second metatarsomere (best seen in dorsal view)	***Mircogioton* Orchymont, 1937**
	(one species known from China: *Mircogioton coomani* Orchymont, 1937)	
–	Mesoventral plate not contacting metaventrite, separated from the latter by a broad gap. First metatarsomere only a little longer than second metatarsomere (best seen in dorsal view)	***Omicrogiton* Orchymont, 1919**
a	Pronotum with fine mesh-like microsculpture on interstices (best seen with spot light and using the light diffuser). Adeagus as in Figs 17–18, with parameres lacking the S-shaped sclerite and median lobe narrow apically	***Omicrogiton coomani* Balfour-Browne, 1939**
–	Pronotum without microsculpture on interstices. Parameres with or without S-shaped sclerite	**b**
b	Aedeagus elongate. Phallobase very short. Paramere without S-shaped strongly sclerotized part, narrow and nearly straight. Median lobe very wide apically, with very large gonopore (Fig. 16)	***Omicrogiton roberti* sp. n.**
–	Aedeagus robust and wide. Phallobase only slightly shorter than parameres. Parameres with strongly sclerotized S-shaped sclerite. Median lobe narrow apically, with small subapical gonopore (Fig. 15)	***Omicrogiton hainanensis* sp. n.**
4	Mesocoxae widely separated; mesoventral plate much wider than long, widely contacting metaventrite. Prothorax with antennal grooves	**5**
–	Mesocoxae rather narrowly separated; mesoventral plate in form a narrowly carinate elevation, only narrowly contacting mesoventrite. Prothorax without antennal grooves	***Paromicrus* Scott, 1913**
	(two species from Taiwan available in our material)	
5	Size 2.0–2.2 mm, highly convex beetles. Elytral series deeply impressed especially sublaterally. Epipleuron wide anteriorly, then becoming extremely narrow, seemingly absent in posterior third of elytra. Pronotum with a transverse series of slightly coarser punctures along posterior margin	***Peratogonus* Sharp, 1884**
	(single species occurring in China, *Peratogonus reversus* Sharp, 1884)	
–	Size 1.2–1.7 mm, at most moderately convex beetles. Elytral series not distinctly impressed. Epipleuron gradually narrowing posteriad, well developed in the posterior third of elytra. Pronotum without distinct transverse row of slightly larger punctures along posterior margin	***Noteropagus* Orchymont, 1919**
	(multiple species occur in China, a taxonomic revision is needed)	

## Species-level taxonomy

### Omicrogiton coomani

Taxon classificationAnimaliaColeopteraHydrophilidae

Balfour-Browne, 1939

Figs 3–4 , 17–18 , 23–24

Omicrogiton coomani Balfour-Browne, 1939: 471.

#### Type material examined.

Lectotype (hereby designated): dissected male (BMNH): “LACTHO / Tonkin. / de Cooman // Andrewes / Bequest / B. M. 1922-22 // *Omicrogiton* / *coomani* Paratypes! / J. Balfour-Browne det. // A. d’Orchymont det. / *Omicrogiton* / *insularis* / d’Orchymont // Coll. d’Orchym.”. Paralectotypes: 1 unsexed spec. (BMNH): same data as the lectotype [this specimen was originally pinned on the same pin as the lectotype, and is now moved to the seperate pin to whoch copies of the aforementioned label are attached]; 1 unsexed specimen (BMNH): “ LACTHO / Tonkin. / de Cooman // C. G. Champion / Brit. Mus. / 1925-42 // A. d’Orchymont det. / *Omicrogiton* / *insularis* / d’Orchymont // PARATYPE // *Omicrogiton* / *coomani* Type! / J. Balfour-Browne det.”; 1 unsexed specimen (BMNH): “ LACTHO / Tonkin. / de Cooman // C. G. Champion / Brit. Mus. / 1925-42 // A. d’Orchymont det. / *Omicrogiton* / *insularis* / d’Orchymont // *Omicrogiton* / *coomani* Paratype! / J. Balfour-Browne det.”.

**Figures 1–6.**
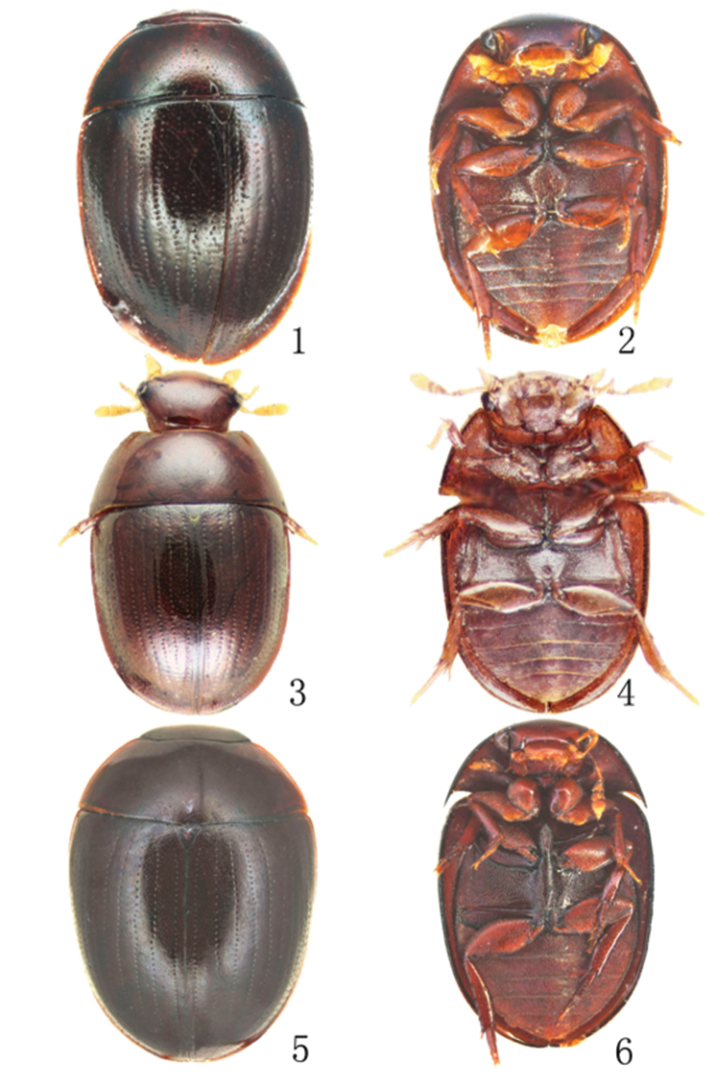
General habitus of Chinese *Omicrogiton* Orchymont, 1919 and *Mircogioton* Orchymont, 1937 **1–2** *Omicrogiton roberti* sp. n. in dorsal and ventral view **3** Lectotype of *Omicrogiton coomani* Balfour-Browne, 1939, dorsal view **4** Paralectotype of *Omicrogiton coomani*, ventral view **5–6** *Mircogioton coomani* Orchymont, 1937, dorsal and ventral view.

#### Additional material examined.

**CHINA: Guangdong**: 3 males, 4 females, 87 unsexed spec. (SYSU): Zhaoqing, Heishiding Natural Reserve, 4–6.x.2013, Fenglong Jia, Ye Jia, Bingjie Chen, Renchao Lin et Weilin Xu leg.; 2 males, 2 females, 15 unsexed spec. (SYSU): Fengkai, Heishiding Natrual Reserve, 179 m, 12.v.2011, Song Keqing leg.; 138 spec. (SYSU): Fengkai, Heishiding Natural Reserve, 20.xi.2010, Fenglong Jia leg.; 88 spec. (SYSU): Fengkai, He’erkou, in decaying banana trunks, 14.viii.2010, Fenglong Jia leg.; 14 spec. (SYSU): Fengkai, Heishiding Natural Reserve, 2.v.2011, Fenglong Jia leg.; 2 males, 1 female, 44 spec. (SYSU): Fengkai, Guangling village, 9.x.2010, Yan Mei, Lijun Yang, Yali Yu leg.; 1 male (SYSU): Fengkai, Yanshuitian, 8.x.2010, Yan Mei et Lijun Yang leg. 1 male, 11 unsexed specimens (NMPC): W of Qixing, Heishiding nature reserve, rotting trunks of banana along the dried-up stream in the primary lowland forest, 190-260 m a.s.l., 23°27.9'N 111°54.3'E, 1.–3.v.2011, Fikáček & Hájek leg. **Hainan**: 1 male (NMPC): Limushan Mts., 19°9.1–9.2'N 109°45–46'N, along the road, 550–750 m a.s.l., rotting banana trunks at the stream in secondary forest, 5.v.2011, Fikáček & Zhao leg. **Hong Kong**: 2 males, 3 females, 25 unsexed spec (AFCD): Hong Kong, Wutongzhai, 27.ix.2013, F.L. Jia, Yingming Lee & Eric Chen leg. **VIETNAM**: 2 males (SYSU): Tonkin, Hoa-Binh, leg. A. de Cooman, with labels “Omicrogiton insularis d’Orchym.” [handwritten] and “En-121415 [En-121416, respectively], Sun Yat-sen University, Biomuseum” [transcript from Chinese]; 1 male, 1 female (IZCAS): Tonkin, Hoa-Binh, leg. A. de Cooman.

#### Diagnosis.

Body length 1.9–2.1 mm, width 1.2–1.3 mm. Head and elytra brown; scapus ca. 3.5× as long as antennomeres 2–5 combined. Interstices of pronotum with fine mesh-like microsculpture; prosternum strongly tectiform. Phallobase ca. half as long as parameres, without distinct manubrium; paramere without strongly sclerotized S-shaped portion, rather wide throughout, weakly sinuate on outer margin, apex semicircular; median lobe slightly narrower than paramere, wide basally, then abruptly narrowing and rather narrow in apical half, apex narrowly rounded, gonopore small, apical.

#### Differential diagnosis.

*Omicrogiton coomani* differs from all other species of the genus except of *Omicrogiton cheesmanae* from New Hebrides by the presence of the fine microsculpture on pronotal interstices. It may be also easily distinguished from all other species of the genus by the morphology of the aedeagus, which lacks the strongly sclerotized S-shaped sclerite of the paramere, and has narrow median lobe with small apical gonopore.

#### Remark.

When Balfour-Browne (1939) checked the material of *Omicrogiton insularis* Orchymont, 1919 deposited in BMNH, he found that the specimens from “Tonkin (Lac Tho)” (i.e. Lac Tho in the Hoa Binh Province in northern Vietnam) differ from those from Engano Island near Sumatra (type locality of *Omicrogiton insularis*) and described them under the name *Omicrogiton coomani*. Since the diagnosis of *Omicrogiton coomani* was very short and it was only presented in the discussion concerning another species of the genus from New Hebrides, the species remained virtually unknown and unrecognized in the collections. The aedeagus of the dissected type specimen of *Omicrogiton coomani* (Fig. 17) really differs from that of *Omicrogiton insularis* and we can therefore confirm that *Omicrogiton coomani* is a separate species.

Within this study, we are showing that *Omicrogiton coomani* may co-occur syntopically with other species of the genus, from which it may be distinguished by male genitalia only. To fix the identity of the species and prevent any future confusion, we are hence designating here the only dissected syntype specimen as the lectotype of *Omicrogiton coomani*. Aedeagus of this specimen is shown in Fig. 17.

#### Biology.

All examined specimens were found in the decaying banana trunks, typically in still standing trunk bases which are decaying after the apical part of the plant was cut or broken.

#### Distribution.

China (Guangdong, Hong Kong), northern Vietnam. New for China.

### Omicrogiton hainanensis sp. n.

Taxon classificationAnimaliaColeopteraHydrophilidae

http://zoobank.org/5FAF1857-A193-4186-A8CF-1CE9260EB15E

Fig. 15

#### Type material.

Holotype: male (SYSU): CHINA: Hainan Isl.: Limushan Mts., 19°9.1–9.2'N, 109°45-46'N, along the road, 550–750 m a.s.l., rotting banana trunks at the stream in secondary forest, 5.v.2011, Fikáček & Zhao lgt. Paratypes: 2 males (SYSU, NMPC): same locality as the holotype; 2 males (NMPC): China: Hainan Isl.: Jianfengling Mts., Tiachi Lake env., Bishu villa, rotting banana trunk at the bank of a drying-up stream in the primary forest above the hotel area, 18°44.7'N 108°50.7'E, 950 m a.s.l., 9–11.v.2011, Fikáček, Kubeček & Li lgt.

#### Diagnosis.

Body length 1.9–2.0 mm. Head and elytra brown; scapus ca. 3.5× as long as antennomeres 2–5 combined. Interstices of pronotum without microsculpture; prosternum weakly tectiform. Phallobase ca. as long as parameres, wide anteriorly, with wide rounded manubrium; paramere with strongly sclerotized S-shaped portion and membranous mesal and apical portions, sclerotized parts of left and right paramere forming very obtuse angle basally; median lobe narrower than phallobase and paramere, rather wide basally, gradually narrowing towards apex, apex rather widely rounded, gonopore subapical (Fig. 15).

#### Description.

***Form and Color.*** Body oval, weakly convex, length 1.9–2.0 mm, width 1.2 mm. Head, pronotum and elytra brown; lateral margin of elytra paler than disc; labrum, maxillary palpomeres and antennomeres 1-6 reddish brown, antennal club of antennae slightly paler; ventral surface brown, legs reddish brown.

***Head.*** Clypeus with fine punctures, interstices without microsculpture; lateral deflexed extensions not defined from clypeal disc by ridge; anterior margin with narrow marginal bead laterally. Frontoclypeal suture undetectable. Frons with sparser and coarser punctures than on clypeus, interstices without microsculpture. Eyes small, clearly protruding, interocular distance ca. 5× as wide as one eye in dorsal view. Labrum exposed, sinuate on anterior margin. Mentum dull, densely granulate, without punctures, ca. 2× as wide as long, not depressed anteromedially, anterior margin slightly protruding medially. Submentum declined below the level of mentum. Antenna with 9 antennomeres, scapus ca. 3.5× as long as antennomeres 2–5 combined, club compact, last club antennomere the widest. Maxillary palpomere 2 moderately swollen, palpomere 4 almost symmetrical, widest at midlength, equal in length to palpomere 2, longer than palpomere 3.

***Thorax.*** Pronotum ca. 2.6× as wide as long. Pronotal punctation similar to that on frons, interstices without microsculpture. Lateral margins with strong bead overlapping to anterior margin, posterior margin of pronotum without bead. Prosternum weakly tectiform; antennal grooves absent. Mesoventrite strongly and abruptly raised medially to form a narrow longitudinal lamina not reaching metaventral process posteriorly; cavities for reception of procoxae absent. Metaventrite weakly convex, without glabrous median portion, with weak posteromedial depression on elevated portion. Elytra widely explanate laterally, with 10 series of large punctures, series 1–5 almost reaching base, series 6–10 abbreviated anteriorly; interval punctures very fine but distinct, similar to those on pronotum, interstices without microsculpture; humeral bulge absent; lateral margin of elytron finely serrate; epipleuron wide throughout. Profemur glabrous, anterior margin angulate near base, with a large basal depression with golden pubescence, tibial groove sharply defined. Mesofemur with sparse and coarse punctures on anterior half, each puncture with a short seta; posterior half glabrous, with fine longitudinal sculpture. Metafemur with fine longitudinal sculpture and scattered fine punctures. Tibiae flat, meso- and metatibiae with long and stout spines along outer face and 1 or 2 pairs of spines on apical half of inner face; metatibial long spur ca. as long as first metatarsomere. First metatarsomere almost as long as metatarsomeres 2–3 combined.

***Abdomen.*** Abdomen with five ventrites; first ventrite not longer than ventrites 2–5 each; first ventrite without median longitudinal carina, fifth ventrite narrowly rounded, not emarginate apically.

***Male genitalia.*** Phallobase ca. as long as parameres, wide anteriorly, with wide rounded manubrium; paramere with strongly sclerotized S-shaped portion and membranous mesal and apical portions, membranous apex of paramere widely rounded; sclerotized parts of left and right paramere forming very obtuse angle basally; median lobe narrower than phallobase and paramere, wide basally, gradually narrowing apicad, apex rather widely rounded, gonopore subapical (Fig. 15).

#### Differential diagnosis.

*Omicrogiton hainanensis* sp. n. belongs to the species with strongly sclerotized S-shaped portion of the paramere, together with the Oriental *Omicrogiton insularis* Orchymont, 1919 and *Omicrogiton gomyi* Bameul, 1986 from the Reunion Island (Bameul 1986). It differs from *Omicrogiton insularis* by the much wider aedeagus (aedeagus is generally very narrow in *Omicrogiton insularis* (Fig. 19), much wider parameres with bases of strongly sclerotized parts forming a very obtuse angle (parameres are narrow and bases of sclerotized portions form acute angle in *Omicrogiton insularis*) and wide median lobe with subapical gonopore (median lobe is extremely narrow apically and the gonopore is situated at midlength in *Omicrogiton insularis*). The aedeagus of *Omicrogiton gomyi* is similar to that of *Omicrogiton hainanensis* in the proportions (i.e., it is wide and robust in both species), but *Omicrogiton gomyi* easily differs by wide median lobe with apical gonopore. *Omicrogiton coomani* and *Omicrogiton roberti*, differ from all above species including *Omicrogiton hainanensis* in parameres lacking the strongly sclerotized S-shaped portion, and *Omicrogiton coomani* and *Omicrogiton cheesmanae* may be distinguished from other species including *Omicrogiton hainanensis* by the pronotum with fine mesh-like microsculpture.

#### Etymology.

The species name is patronymic, referring to the Hainan Island where this species is commonly collected.

#### Biology.

All type specimens were collected in decaying banana trunks in primary or secondary rainforests. On the type locality, the specimens of this species were collected in the same banana trunk as two other *Omicrogiton* species occurring in Hainan (i.e. *Omicrogiton roberti* sp. n. and *Omicrogiton coomani*), which indicates that multiple species may occur syntopically in this genus. For this reason, we excluded females from the type series of this species.

#### Distribution.

China (Hainan).

### Omicrogiton roberti sp. n.

Taxon classificationAnimaliaColeopteraHydrophilidae

http://zoobank.org/960DC7E9-66F4-423B-AC47-0349093381C9

Figs 1 , 2 , 16

#### Type material.

Holotype: male (SYSU): CHINA: Hainan isl., Limushan Mts., Limu temple, 5.v.2011, 19°9.1–9.2'N, 109°45–46'E, 550–750 m; along the road, rotting banana trunks at the stream in secondary forest, Fikáček & Zhao lgt.

#### Diagnosis.

Body length 2.1 mm. Head and elytra black or dark brown; pronotum paler than head and elytra. Scapus ca. 2.5× as long as antennomeres 2–5 combined. Prosternum strongly tectiform, with low longitudinal carina medially. Phallobase much shorter than parameres, with thin and long basal manubrium; paramere without distinct S-shaped more sclerotized portion, narrow, weakly curved on outer margin, rounded apically; median lobe much broader than paramere, bottle-shaped, widest at basal third, strongly narrowing ca. at midlength, apex broadly truncate; gonopore large, situated subapically (Fig. 16).

#### Description.

***Form and Color.*** Body oval, weakly convex (Fig. 1), length 2.1 mm, width 1.35 mm. Head and elytra dark brown; pronotum brown; lateral margin of elytra paler than disc; labrum, maxillary palpomeres and antennomeres 1–6 reddish brown, antennal club paler; ventral surface brown, legs reddish brown.

***Head.*** Clypeus with rather densely arranged fine punctures, interstices without microsculpture; lateral deflexed extensions not defined by ridge. Anterior margin of clypeus with narrow bead laterally. Frontoclypeal suture undetectable. Frons with sparser and coarser punctures than on clypeus, without microsculpture on interstices. Eyes small, clearly protruding, interocular distance ca. 7× as wide as one eye in dorsal view. Labrum exposed, sinuate on anterior margin. Mentum densely granulated, without punctures, ca. 2× as wide as long, not depressed anteromedially, anterior margin slightly protruding medially. Submentum below the level of mentum. Antenna with 9 antennomeres, scapus ca. 2.5× as long as antennomeres 2–5 combined, club compact, last club antennomere the widest. Maxillary palpomere 2 moderately swollen, palpomere 4 almost symmetrical, widest in middle, equal to palpomere 2 in length, longer than palpomere 3.

***Thorax.*** Pronotum ca. 2.6× as wide as long; pronotal punctation similar to that on frons, interstices without microsculpture. Lateral margins with strong bead overlapping to anterior margin, posterior margin of pronotum without bead. Prosternum strongly tectiform, antennal grooves absent. Mesoventrite strongly and abruptly raised medially to form a narrow longitudinal lamina not contacting metaventral process posteriorly, cavities for reception of procoxae absent. Metaventrite weakly convex, with a small glabrous portion, with a posteromedial depression on elevated portion. Elytra widely explanate laterally, with 10 series of large punctures, series 1–5 almost reaching base, series 6–10 abbreviated anteriorly; interval punctures very fine but distinct, similar to on pronotum; interstices without microsculpture. Humeral bulge absent, lateral margin of elytron very finely serrate; epipleuron wide throughout.Profemur glabrous, anterior margin angulate near base, with a large basal depression with golden pubescence, tibial groove sharply defined. Mesofemur with sparse and strong punctures on anterior half, each puncture with a short seta; posterior half glabrous, with fine longitudinal sculpture. Metafemur with fine longitudinal sculpture and scattered fine punctures. Tibiae flat, meso- and metatibiae with long and stout spines along outer face and 1 or 2 pairs of spines on apical half of inner face; metatibial long spur longer than first tarsomere. First metatarsomere almost as long as metatarsomeres 2–3 combined.

***Abdomen.*** Abdomen with five ventrites; first ventrite not longer than ventrites 2–5, without median longitudinal carina; fifth ventrite rounded, not emarginate apically.

***Male genitalia.*** Phallobase much shorter than parameres, with a thin and long basal manubrium. Paramere without S-shaped strongly sclerotized portion, narrow throughout, weakly curved on outer margin, rounded apically. Median lobe much broader than paramere, bottle-shaped, widest in basal third, strongly narrowed ca. at midlength, apex broadly truncate; gonopore large, situated subapically (Fig. 16).

#### Different diagnosis.

*Omicrogiton roberti* is similar to *Omicrogiton coomani* Balfour-Browne, 1939 in the aedeagus without the strongly sclerotized S-shaped portion of the paramere. It differs from *Omicrogiton coomani* by the morphology of the aedeagus (median lobe very wide with very large subapical gonopore and very short phallobase in *Omicrogiton roberti*, rather narrow and with small apical gonopore and rather long phallobase in *Omicrogiton coomani*) and by the pronotal interstices without fine mesh-like microsculpture (with fine mesh-like microsculpture in *Omicrogiton coomani*).

#### Etymology.

The species is named after Dr. Robert Bagrie Angus, a British specialist on the Helophoridae, who helped us a lot with this study.

#### Biology.

The holotype was collected in a decaying banana trunk together with specimens of *Omicrogiton hainanensis* and *Omicrogiton coomani*.

#### Distribution.

China (Hainan).

### Mircogioton coomani

Taxon classificationAnimaliaColeopteraHydrophilidae

Orchymont, 1937

Figs 5–6 , 14 , 20

Mircogioton coomani Orchymont, 1937: 464.
Ischyromicrus cognitus Malcolm, 1981: 267. **New synonym.**
Mircogioton cognitus (Malcolm); Hansen 1991: 226

#### Material examined.

**CHINA, Yunnan**: 2 males, 6 unsexed spec. (SYSU, NMPC): Laiyanghe, Xinzhai Cun, 1487 m, 22.631°N, 101.132°E, 21.v.2011, Song Keqing lgt.; 1 male, 2 unsexed spec (SYSU): Mandian Nabanhe Conv., 11.i.2004, Li & Tang lgt.; 1 spec. (NMPC): Laiyanghe, Yutang village, in decaying banana trunk, 22.v.2011, Keqing Song lgt. **VIETNAM**: 1 male (IZCAS): Tonkin, Hoa-Binh, leg. A. de Cooman.

#### Diagnosis.

Body length 3.2–3.4 mm, width 2.2 mm. Dorsal surface dark brown, ventral surface brown to dark brown. Labrum weakly bisinuate on anterior margin, not distinctlx projecting anteriad. Scapus ca. 2.2× as long as antennomeres 2–5 combined, slightly shorter than antennal club. Head, pronotum and elytra with similar sparse and fine punctation, interstices without fine microsculpture; elytra with 10 series of punctures, series 6–7 abbreviated anteriorly. Prosternum strongly tectiform, sharp anteriorly. Mesoventral elevation much longer than wide, with distinct longitudinal groove medially, posteriorly fused with metaventral process, not projecting posteriad into a process overlapping metaventrite. Metaventrite with a longitudinal glabrous elevated band medially, forming together with mesoventral plate a joint meso-metaventral elevation. Phallobase ca. 0.3× as long as paramere; paramere rather wide throughout, outer margin slightly concave subapically, apex semicircular. Median lobe slightly narrower than paramere, lateral margin almost parallel, apex narrowly rounded, gonopore of moderate size, subapical (Fig. 20).

#### Differential diagnosis.

*Mircogioton coomani* differs from *Mircogioton spinosus* Bameul, 1993, *Mircogioton seriatus* Hebauer, 2006 and *Mircogioton irregularis* Hebauer, 2006 in mesoventrite fused with metaventral process (in contrast, mesoventrite is projecting into a long process overlapping metaventrite in the latter three species). It differs from *Mircogioton grandis* Bameul, 1993 and *Mircogioton julieae* (Malcolm, 1981) by the anterior margin of the labrum bisinuate (in contrast, labrum is simply concave on anterior margin in the latter two species). From *Mircogioton julieae* it also differs by apically broad paramere and apex of median lobe not distinctly narrowed. From *Mircogioton grandis* it may be also distinguished by smaller body size (up to 3.5 mm, in comparison with 3.8 mm in *Mircogioton grandis*).

#### Remark.

This species was described by d’Orchymont (1937) based on a single female collected by A. de Cooman in “Tonkin, Hoa Binh”. The senior author examined one male collected by the same collector and bearing the same label data. Except of clearly being a part of the same material from which *Mircogioton coomani* was collected, the examined specimen agrees in all details with the original description. We therefore consider it represents *Mircogioton coomani* although we have not checked the female holotype.

Malcolm (1981) described *Ischyromicrus cognitus* Malcolm, 1981 based on a female from upper Mekong (later transferred to *Mircogioton* by Hansen (1991)). The species was redescribed by Bameul (1993). The characters described by Malcolm (1981) and Bameul (1993) are identical with the specimens of *Mircogioton coomani* in our hands. The type locality of *Mircogioton cognitus* is situated in northeastern Laos not far from the border with China rather than in Vietnam as supposed by Malcolm (1981) (Bameul 1993, Hansen 1999). Bameul (1981) moreover noticed that “in the description of *Mircogioton cognitus*, no characters really differ from those given by d’Orchymont (1937) in his description of *Mircogioton coomani*” and supposed that *Mircogioton cognitus* is synonym of *Mircogioton coomani*. We are following this opinion here and consider *Mircogioton cognitus* as a junior subjective synonym of *Mircogioton coomani*.

#### Biology.

The recently collected specimens examined here were found in decaying banana trunk (K.-Q. Song and L. Tang, pers. comm.).

#### Distribution.

China (Yunnan), northern Laos. New genus and species for China.

### Peratogonus reversus

Taxon classificationAnimaliaColeopteraHydrophilidae

Sharp, 1884

Figs 8–12 , 21–22

Peratogonus reversus Sharp, 1884: 461.

#### Type material examined.

Syntypes: 2 specimens on one card (BMNH): “*Peratogonus reversus* Type DS Nagasaki 14.4.81 // Japan G. Lewis Sharp coll. 1905 – 313”; 1 upside down spec. (BMNH): “*Peratogonus reversus* Sharp // Japan G. Lewis // 30.3.81”.

**Figures 7–13.**
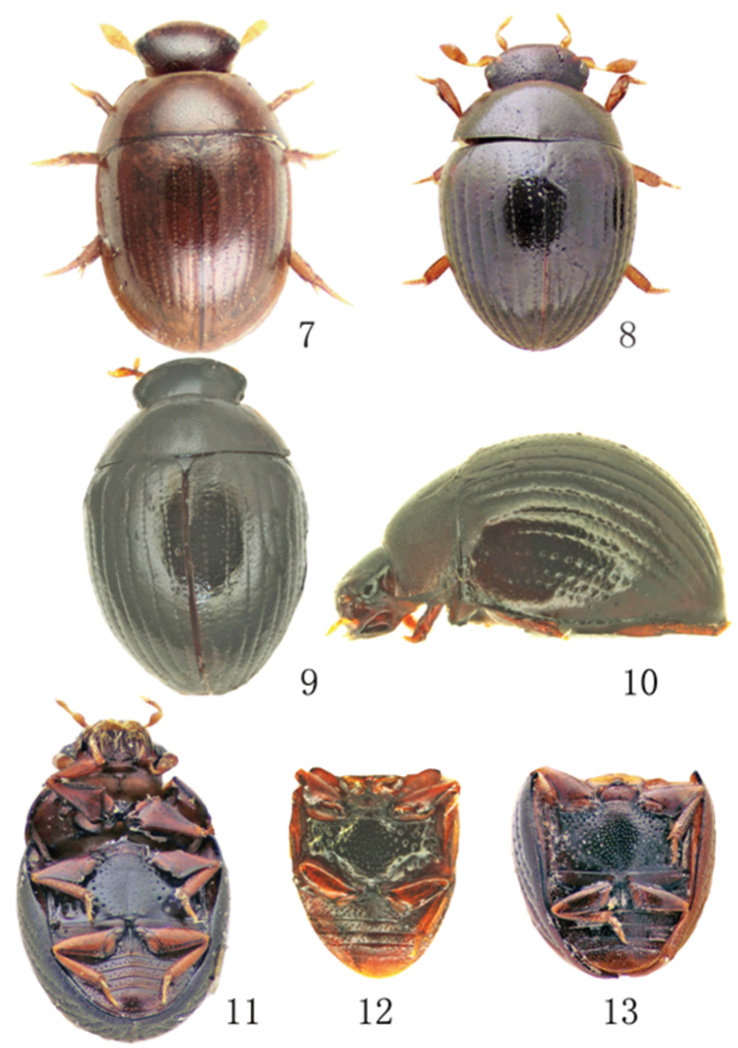
Morphology of *Omicrogiton* Orchymont, 1919 and *Peratogonus* Sharp, 1884 **7** Dorsal habitus of *Omicrogiton insularis* Orchymont, 1919 **8–11** Habitus of *Peratogonus reversus* Sharp, 1884 (**8** syntype from Japan, dorsal view **9** specimen from China, dorsal view **10** specimen from China, lateral view **11** syntype from Japan, ventral view) **12–13** Comparison of metaventral punctation of *Peratogonus* (**12** *Peratogonus reversus* **13** *Peratogonus grandis* Bameul, 1994).

#### Additional material examined.

**JAPAN**: 1 male (BMNH): “Japan, Kobe. Mayasan 14.vi.29 JEA Lewis. Nr 1530”; 1 male (NMPC): Kanagawa Pref., Manazuru Peninsula, 4.xi.2006, P. Jaloszynski lgt.; 1 spec. (NMPC): Chiba Pref., Kôzaki shrine, Kôzaki-machi, 15.x.2001, P. Jaloszynski lgt. **CHINA: Guangdong**: 1 male (SYSU): Conghua, Liuxihe forest park, 16.v.2012, Tong Xiaoli leg. (in Chinese); 1 male (SYSU): Fengkai, Heishiding Natural Reserve, 23°27.9'N 111°54.3'E, 190–260 m, Fenglong Jia leg.; 1 male (NMPC): W of Qixing, Heishiding nature reserve, sifting of moist leaf litter in the dried-up streambeds and along the streams in the primary lowland forest, 190–260 m a.s.l., 23°27.9'N, 111°54.3'E, 1.–3.v.2011, Fikáček & Hájek lgt. **Jiangxi**: 1 male (NMPC): Jinggangshan Mts., Xiangzhou (forested valley S of the village), cut and decaying tops of bamboo trunks at side of a trail in the secondary forest and among the fields, 26°35.5'N, 114°16.0'E, 374 m, 26.iv.2011, Fikáček & Hájek lgt. **Taiwan**: 1 male, 9 spec. (NMPC, SYSU): Maoli County, Nanjhuang Twnsh., S-Nanjhuang Rd. 124, km 3 + forest road, forest compost, 26.x.2010, S. Vít lgt.

#### Diagnosis.

Body length 2.1 mm, width 1.5 mm, strongly convex. Head and pronotum with fine microsculpture between punctures. Elytra with 10 striae, striae 1–5 reaching elytral base, striae 6–10 abbreviated anteriorly, not reaching base; elytral intervals with distinct fine punctures, without microsculpture between punctures. Prosternum steeply raised in middle to form a triangular medially carinate tablet. Mesoventrite flat medially, widely fused with metaventrite. Metaventrite laterally with much coarser and stronger punctures than on its median portion. Aedeagus (Figs 21–22) with phallobase ca. as long as paramere, tube-like; paramere broad basally, gradually narrowing towards apex; median lobe slender, parallel-sided, with very long basal struts, gonopore indistrinct, apex slightly emarginate.

**Figures 14–22.**
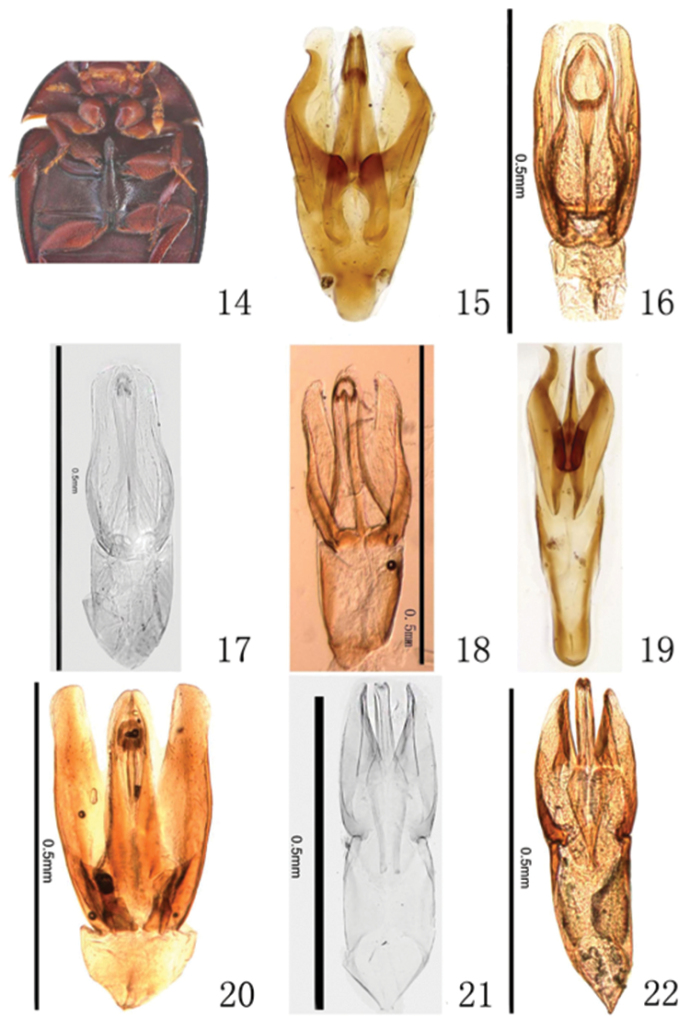
Ventral morphology (14) and morphology of aedeagus (15–22). **14** detail of meso- and metaventrite of *Mircogioton coomani* Orchymont, 1937 **15** *Omicrogiton hainanensis* sp. n., holotype **16** *Omicrogiton roberti* sp. n., holotype **17–18** *Omicrogiton coomani* Balfour-Browne, 1939 (**17** lectotype; **18** non-type specimen from China) **19** *Omicrogiton insularis* Orchymont, 1919, specimen from Sarawak **20** *Mircogioton coomani* Orchymont, 1937, Chinese specimen **21–22** *Peratogonus reversus* Sharp, 1884 (**21** syntype **22** specimen from China).

#### Differential diagnosis.

This species can be easily distinguished from *Peratogonus grandis* Malcolm, 1981 occurring in India (Sikkim) by punctures on the lateral portion of the metaventrite much deeper and larger than medially. From *Peratogonus corporaali* Orchymont, 1926 occurring in Indonesia (Java), it may be distinguished by pronotum with distinct microsculpture between punctures, elytra with striae 6–10 not reaching elytral base (stria 8 almost reaching base, striae 9–10 reaching base in *Peratogonus corporaali*), elytral intervals with distinct punctures, flat mesoventral plate, and metaventrite with coaser and sparser punctures medially.

#### Remark.

This species was firstly described from Nagasaki, Kyushu in southern Japan by Sharp (1884). It was subsequently reported from Taiwan by Knisch (1921). The comparison of the specimens from Taiwan and southern continental China revealed that they are identical with those from Japan.

When Malcolm (1981) described *Peratogonus grandis* Malcolm, 1981, he diagnosed it from *Peratogonus reversus* by the different body size (2.21×1.64 mm in *Peratogonus grandis* versus 1.72×1.31 in *Peratogonus reversus*) and by the shallower and smaller punctures on lateral portion of the metaventrite. The material examined by us revealed that the specimen of *Peratogonus reversus* examined by Malcolm (1981) was smaller than its individuals usually are (i.e. body length 1.9–2.2 mm). Therefore, the body size can not be used as a reliable character to distinguish the two species, in contrast to the punctation of the metaventrite, which seems to be a reliable character to distinguish the two species.

#### Biology.

Most specimens examined here were found by sifting forest leaf litter.

#### Distribution.

China (Guangdong, Jiangxi, Taiwan), Japan (Honshu, Kyushu). New genus and species for mailand China.

**Figures 23–24.**
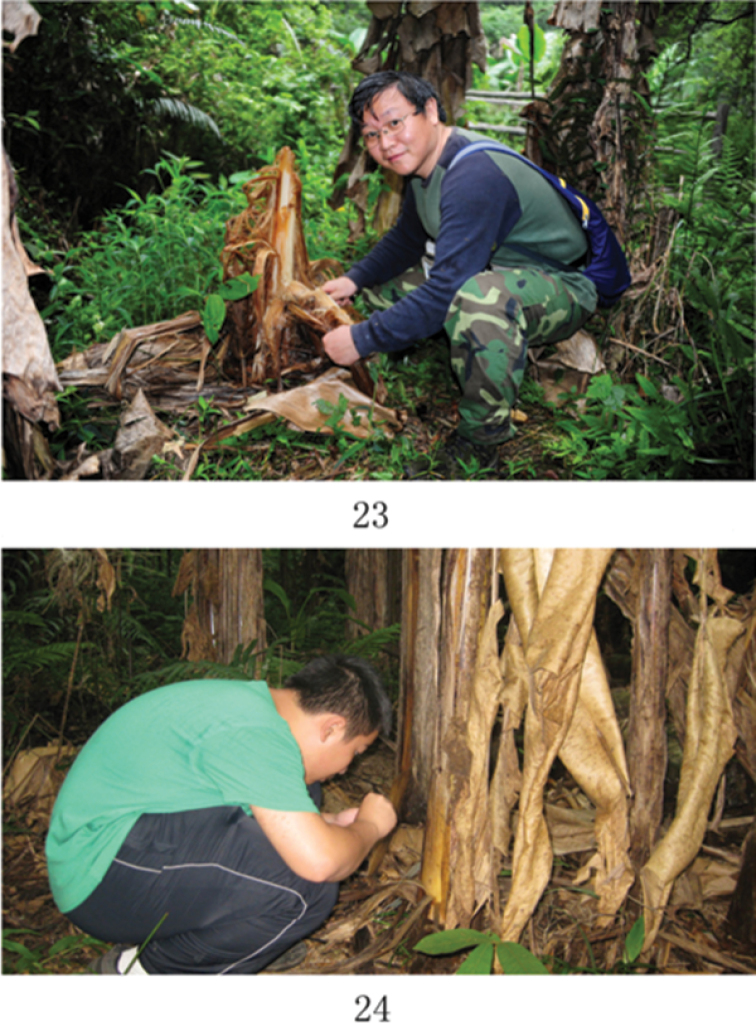
Examples of habitats of Chinese *Omicrogiton* Orchymont, 1919.

## Supplementary Material

XML Treatment for Omicrogiton coomani

XML Treatment for Omicrogiton hainanensis

XML Treatment for Omicrogiton roberti

XML Treatment for Mircogioton coomani

XML Treatment for Peratogonus reversus
